# Fabrication and characterization of a polysulfone-graphene oxide nanocomposite membrane for arsenate rejection from water

**DOI:** 10.1186/s40201-015-0217-8

**Published:** 2015-08-22

**Authors:** Reza Rezaee, Simin Nasseri, Amir Hossein Mahvi, Ramin Nabizadeh, Seyyed Abbas Mousavi, Alimorad Rashidi, Ali Jafari, Shahrokh Nazmara

**Affiliations:** Department of Environmental Health Engineering, School of Public Health, Tehran University of Medical Sciences, Tehran, Iran; Center for Water Quality Research, Institute for Environmental Research, Tehran University of Medical Sciences, Tehran, Iran; Center for Solid Waste Research, Institute for Environmental Research, Tehran University of Medical Sciences, Tehran, Iran; National Institute of Health Research, Tehran University of Medical Sciences, Tehran, Iran; Department of Chemical and Petroleum Engineering, Sharif University of Technology, Tehran, Iran; Nanotechnology Research Center, Research Institute of Petroleum Industry (RIPI), Tehran, Iran; Department of Environmental Health Engineering, School of Public Health, Lorestan University of Medical Sciences, Khorramabad, Iran

**Keywords:** Mixed matrix membrane, Polysulfone, Graphene oxide, Hydrophilicity, Arsenate

## Abstract

**Background:**

Nowadays, study and application of modified membranes for water treatment have been considered significantly. The aim of this study was to prepare and characterize a polysulfone (PSF)/graphene oxide (GO) nanocomposite membrane and to evaluate for arsenate rejection from water.

**Materials and methods:**

The nanocomposite PSF/GO membrane was fabricated using wet phase inversion method. The effect of GO on the synthesized membrane morphology and hydrophilicity was studied by using FE-SEM, AFM, contact angle, zeta potential, porosity and pore size tests. The membrane performance was also evaluated in terms of pure water flux and arsenate rejection.

**Results:**

ATR-FTIR confirmed the presence of hydrophilic functional groups on the surface of the prepared GO. FE-SEM micrographs showed that with increasing GO content in the casting solution, the sub-layer structure was enhanced and the drop like voids in the pure PSF membrane changed to macrovoids in PSF/GO membrane along with increase in porosity. AFM images indicated lower roughness of modified membrane compared to pure PSF membrane. Furthermore, contact angle measurement and permeation experiment showed that by increasing GO up to 1 wt%, membrane hydrophilicity and pure water flux were increased. For PSF/GO-1, pure water flux was calculated about 50 L/m^2^h at 4 bar. The maximum rejection was obtained by PSF/GO-2 about 83.65 % at 4 bar. Moreover, it was revealed that arsenate rejection depended on solution pH values. It was showed that with increasing pH, the rejection increased.

**Conclusions:**

This study showed that application of GO as an additive to PSF casting solution could enhance the membrane hydrophilicity, porosity, flux and arsenate rejection.

## Background

In recent years, a growing public concern has arisen over release of toxic pollutants such as inorganic ions, metals and synthetic organic matters into the water due to increasingly industrial and agricultural activities. Among these toxicants, arsenic is a serious threat in water resources of some regions [1, 2]. Toxicological and epidemiological studies proven that inorganic arsenic could cause carcinogenic and non-carcinogenic effects in human [3]. World health organization (WHO) and united state protection agency (USEPA) had classified arsenic as class A carcinogens list [4]. International agency for research on cancer (IARC) also classified inorganic form of arsenic in class I carcinogens list [5]. With regard to strict regulations for control and removal of arsenic in drinking water, and limitations of conventional water treatment processes (e.g. generation of toxic intermediates and low efficiencies) looking for new technologies is of great interest [3, 6]. Membrane process can be considered as a promising technology for arsenic removal due to its several advantages such as no need to add chemicals, no generation of sludge, ease of system capacity development, separation in continuous mode, ease of integration with other processes, minimum dependency to environmental conditions and capable of microorganisms and solutes removal [7, 8]. However, certain drawbacks associated with common membranes are low water recovery, fouling problem and high-energy consumption [9, 10]. In recent years to overcome these drawbacks, different studies have been conducted in order to modify polymeric membranes to enhance the permeability, rejection and decreasing fouling problem and reduce the investment and operational costs [11]. Accordingly, various works such as physical blending, plasma treatment, polymer grafting and chemical reactions have been carried out to modify the membranes [12, 13]. Among these methods physical blending is preferred due to the simplicity procedure using phase inversion technique [14]. Physical blending consist of mixing of polymeric materials with inorganic nanoparticles (e.g. TiO_2_ [15], ZnO [16], silica [17]) and recently carbon allotropes [11, 18, 19]. Adding inorganic nanoparticles to membrane matrix can enhance the membrane hydrophilicity, strength, permeability and antifouling characteristics [18, 20]. Graphene and its derivatives due to unique two-dimensional structure, one-atom-layer-thick, high theoretical surface area (2630 m^2^/g), good mechanical properties, non-harmful effects, low cost production have attracted interest for different application especially polymeric membrane modification [21, 22]. Graphene oxide (GO) is also highly hydrophilic due to presence of oxygen containing functional groups (e.g. hydroxyl, carboxyl, carbonyl and epoxy) [12, 23]. When thin sheets of carbon atoms (GO) are added to a polymer matrix at low content and proper procedure, it could significantly improve the physical properties of the base polymer [21, 24]. Among different synthetic polymers, polysulfone (PSF) is the one that is widely used for various membranes fabrication such as filtration, ultrafiltration, hemodialysis and bioreactor technologies [13, 25]. The reasons for wide use of this type of polymer are good characteristics such as desire mechanical and thermal properties, high chemical stability, high resistance in a wide range of pH and high solubility in a broad range of polar solvents (dimethylformamide, dimethylacetamide, dimethylsulfoxide) [13, 21, 25]. One of the main drawbacks of PSF membrane is fouling problem and consequently reduction of the membrane lifetime. Actually, this type of membrane is influenced by fouling problem more than other membrane materials because of the hydrophobic nature of the membrane and interactions between the membrane surface charges and the foulants [13, 21]. A few studies have used GO in casting solution to improve the water permeability, antifouling properties and mechanical strength characteristics of the mixed matrix membrane. Zhao et,al showed that synthesized PVDF/GO ultrafiltration membrane had higher pure water flux compared to PVDF due to improvement of the surface hydrophilicity [12]. Wang et, al also reported that GO nanosheet as a hydrophilic modifier could enhance the water flux of the fabricated ultrafiltration membrane with an improvement in the antifouling property [14]. In another study, Zinadini et.al showed that water permeability, hydrophilicity and antifouling properties of the PES/GO membrane were enhanced compared to pure PES membrane [26]. Xia et, al also revealed that employment of a certain amount of GO in the matrix could improve the water flux, hydrophilicity and antifouling characteristics of a type of synthesized PVDF/GO membrane used for natural organic matter removal [27]. The aim of this study is to synthesis and characterizes a PSF/GO nanocomposite membrane in order to reject arsenic from water. In this work, GO was applied to PSF matrix as a hydrophilic agent. The performance of the synthesized membranes was evaluated by pure water flux measurement and arsenate rejection.

## Materials and methods

### Materials

All chemicals used in the experiments were of reagent grade. Graphite fine powder extra pure (with a mean particle size of <50 μm) was purchased from Merck- Germany. PSF (with average M_w_ = 22,000 g/mol) was obtained from Sigma-Aldrich Co-Germany. N,N-Dimethylformamide >(DMF) was purchased from Sigma-Aldrich and used without purification as a solvent to prepare cast solution. Analytical grade H_2_SO_4_ (98 %-Merck), NaNO_3_ (99 %, Sigma–Aldrich), KMnO_4_ (99 %, Sigma–Aldrich) and H_2_O_2_ (30 % solution, stabilized-Merck) were used as received. Sodium arsenate dibasic heptahydrate (Na_2_HAsO_4_-7H_2_O) was obtained from Sigma-Aldrich. The deionized (DI) water was used in the sample preparation and for pure water flux measurements.

### Preparation and characterization of graphene oxide (GO)

In this study GO was prepared using modified Hummer’s method [28]. Firstly, 5 g graphite powder and 2.5 g sodium nitrate were added to a 500-ml neck flask containing 120 ml concentrated sulfuric acid in ice bath and thoroughly mixed for 30 min. Then under vigorous mixing, 15 g KMnO_4_ was slowly added to the suspension and mixing was continued for 30 min. The rate of adding was controlled to maintain temperature of the reaction below 20 °C. After that, ice bath was removed and the mixture was stirred overnight at room temperature. By elapsing the time, the mixture changed in to sticky and the color changed to brown. Then under mixing condition, 150 ml distilled water was slowly added to the mixture. The temperature was rapidly increased to 98 °C and the color turned to yellow. This aqueous suspension was stirred at 98 °C for 24 h. In order to remove KMnO_4_, 50 ml H_2_O_2_ (30 %) was added to the liquid mixture. For more purification, the liquid mixture of GO was washed by HCL (5 %) and DI water and centrifuged for several times to reach the pH to natural range. Finally, for exfoliating the product, sonication was conducted for 1 h. Then it was filtered and dried in a vacuum oven (at 40 °C for 24 h) to obtain a grey color GO nanoplate powder. Raman spectra of the GO was obtained in the spectral range of 100-4200 cm^-1^ and with 532 nm wavelength incident laser light (Almega Thermo Nicolet Dispersive Raman Spectrometer, Germany). The measurements of the attenuated total reflectance fourier transform infrared spectroscopy (ATR-FTIR) of the GO was performed using a ATR-FTIR spectroscopy in the range between 600 cm − 1 and 4000 cm^−1^ (Tensor 27, Bruker Inc., Germany).

### Fabrication of PSF/GO nanocomposite membrane

In present work, PSF/GO nanocomposite membrane was fabricated via common phase inversion method [14, 29]. For this purpose, PSF was used as bulk material, DMF as solvent, GO nanoplate as the additive and hydrophilic modifier, DMF as the solvent and DI water as the nonsolvent in coagulation bath. The casting solution consist of PSF = 15 %wt, DMF = 85 wt% and GO = (0-0.5-1-2 wt% PSF). PSF and GO powder were dried in vacuum oven at 60 °C for 4 h. At first, four different amounts of GO were dispersed in DMF and was sonicated for 1 h to obtain a homogenous casting solution. Then, under continuous stirring condition, PSF was added to GO/DMF mixture and was allowed to stir for 24 h. Then the casting solution was maintained in room temperature for 24 h without stirring. Finally, casting solution was sonicated to remove remaining air bubbles. The prepared casting solution was casted uniformly onto a smooth and clean glass plate using a casting knife at a thickness of 200 μm. The casted film on the glass was left for air exposure (20 s) followed by immersing into the nonsolvent coagulation bath (DI water at 25 °C). The glass plate was kept in the coagulation bath for 10 min to guarantee complete phase inversion process. Finally the peeled off synthesized membrane was washed with DI water for several times until all the residual solvent removed. The membranes were kept in DI water for characterization and experiments. The synthesized membrane based on GO content named pure PSF, PSF/GO-0.5, PSF/GO-1 and PSF/GO-2.

### Characterization of the prepared membranes

The structure and surface morphology of the membranes were evaluated using a field-emission scanning electron microscope (FE-SEM, S-4160, Hitachi, Japan). For sample preparation, membrane were cut into small pieces and washed with distilled water. For obtaining a good cross section image, the wet pieces were immersed in liquid nitrogen for 1 min to freeze. The frozen pieces of the membranes were fractured and kept in air to dry. The dried samples were coated with a thin layer of gold to increase the electric conductivity before FE-SEM imaging. Atomic force microscopy (AFM) was applied for top surface morphology and roughness analysis. Thermo microscopes Auto probe CP Research (Veeco Instruments, Sunnyvale, CA, USA) was used for AFM analysis. The samples were cut into small pieces (1 cm × 1 cm), washed with distilled water and dried in room temperature. In this study, surface hydrophilicity changes of different fabricated membranes were determined via the contact angle and Zeta potential. The contact angle was analyzed using a water contact angle measurement (OCA 15 Plus, Dataphsycs, Germany). Before contact angle measurement, the samples were dried in oven at 50 °C for 4 h. For more accuracy in the determination of contact angle, 5 different top surface points were measured and the average was reported. The zeta potentials of fabricated membrane were measured by streaming potential method using Electro kinetic Analyzer (EKA, Anton Paar GmbH, Austria) equipped with plated sample cell. For this purpose membrane were cut in 5 cm × 5 cm pieces and zeta potentials were measured at 26 °C and pH of 7. In this measurement method, 0.001 M KCl solution was applied as electrolyte and zeta potential were measured in triplicate for each membrane.

### Membrane porosity and pore size

To evaluate the effect of GO on the membrane structure, the porosity, as the ratio of the volume of voids to the total volume of membrane, was measured using a gravimetry method. For this, the membranes were dried in an air-circulating oven at 50 °C for 24 h. Then the samples were cut into small pieces (1 cm × 1 cm) (5 pieces for each membrane) and weighted. The pieces were immersed in distilled water for 24 h at 25 °C. After removing the droplets on the surface of membrane by a paper filter, the membrane was weighted again. The average of dry and wet weights for each membrane was recorded and the porosity (ε) was calculated using the gravimetry equation 1 [18, 30, 31].

1 $$ \varepsilon \kern0.5em =\kern0.5em \frac{\frac{W_1-{W}_2}{\rho_w}}{\frac{W_1-{W}_2}{\rho_w}+\frac{W_2}{\rho_m}}\kern0.5em \times \kern0.5em 100\% $$

Where, *W*_*1*_ and *W*_*2*_ are wet and dry weights of membrane respectively (g), *ρ*_*w*_ is the density of distilled water (0.998 g/mL) and *ρ*_*m*_ is the density of polymer (PSF = 1.24 g/mL at 25 °C). The average pore radius (r_m_) of the membranes was calculated by following equation known as Guerout–Elford–Ferry equation (Eq. 2) [18, 32].

2 $$ rm\kern0.5em =\kern0.5em \sqrt{\frac{\left(2.9-1.75\varepsilon \right)\kern0.5em \times \kern0.5em 8\eta lQ}{\varepsilon \kern0.5em \times \kern0.5em A\kern0.5em \times \kern0.5em \varDelta P}} $$

where *rm* is the mean pore radius (m), *η* is the water viscosity (8.9 × 10^−4^ Pa · s), *l* is the membrane thickness (m), Q is the volume of the permeate water per unit time (m^3^/s), *A* is the effective area of the membrane (m^2^) and *ΔP* is the operational pressure (Pa).

### Permeation tests and arsenate rejection experiments

**Fig. 1:**
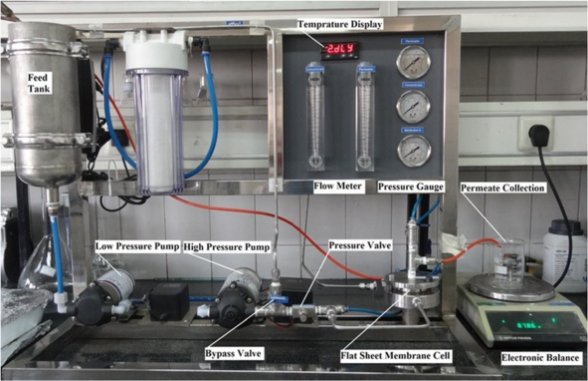
Membrane filtration system used in experiment

In this study, to evaluate the permeation flux and arsenate rejection by fabricated nanocomposite membrane, a lab-scale filtration system was used at dead end mode operation. The main components of the filtration system include a 2-L feed tank (equipped with mixer and temperature control), low and high pressure feed pumps (1 to 15 bar), stainless steel flat membrane module (effective area of 9.6 cm^2^), valves and pressure gauges (Fig. 1). For flux measurements, the membranes were first immersed in distilled water for 24 h. Then the membranes were compacted under 7 bar of distilled water at 25 ± 0.5 °C for 30 min until a constant flux was achieved. Immediately the pressure was reduced to 4 bar and pure water flux test was conducted for 1 h with collecting and measuring the filtrate volume at 5 min intervals. Finally, the flux was calculated using equation 3 [11, 33].

3 $$ {J}_w=\frac{V}{A\varDelta t} $$

Where *J*_*w*_ is the pure water flux (L/m^2^h), *V* is the volume of permeated pure water (L), *A* is the effective area of membrane (m^2^) and *Δt* is the sampling time (h).

after measuring the pure water flux, arsenate sodium solution with initial concentration of 300 ± 10 μg/L was prepared based on a standard procedure [34]. The rejection of arsenate was evaluated at 4 bar. The permeate was collected each 20 min for arsenate analysis, finally the average was reported. Arsenic concentrations was measured by inductively coupled plasma optical emission spectroscopy (HG-ICP/OES) (Model Spectro arcos, Specro Inc, Germany) connected to a hydride generator. The percentage of rejection was calculated using equation 4 [26].

4 $$ \%R\kern0.5em =\kern0.5em \left(1-\frac{C_p}{C_f}\right)\kern0.5em *\kern0.5em 100 $$

Where R is the rejection of arsenate (%), and C_p_ and C_f_ are the concentrations of arsenate in the permeation and feed solution, respectively (μg/L). All pure water flux and rejection experiments were performed in triplicate.

## Results and discussion

### Characterization of graphene oxide

**Fig. 2:**
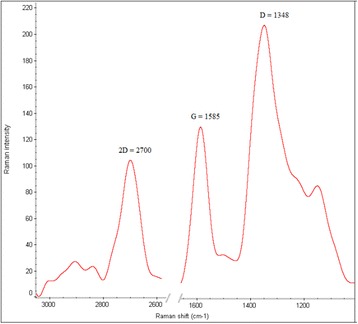
Raman spectrum of synthesized GO

Figure 2 depicts the Raman spectrum of synthesized GO. From the Figures D, G and 2D appeared at 1348, 1585 and 2700 cm^-1^ as GO known peaks [35]. Generally, in graphene Raman spectrum, D band indicates the disordered and defect in graphene structure, G band shows that normal structure of graphene and 2D band is related to number of layers. Graphene Raman spectrum from a single layer and a few layer graphene consist of peak G at around 1580 cm^-1^ and peak 2D at around 2700 cm^-1^. In GO Raman spectrum, as well as G and 2D peaks, typical D band is obvious which appears at around 1350 cm^-1^. This peak (D) is absent in ordered graphene, while in GO, presence of D band is assigned to the developed defect structure in due to oxygen containing functional groups(e.g. hydroxyl) at the edge of graphene plates. As the intensity of peak D is higher, the sample has higher disordered structure. In addition, Raman spectrum can be used for analysis of graphene quality and determination of the layers (up to 5 layers) through the 2D peak shape, width and position. With increase in number of the layers, 2D peak shifts to higher wavelengths and will broaden [36, 37]. In this study based on appeared peaks from Raman spectrum analysis confirmed a few-layer structure of GO.

**Fig. 3:**
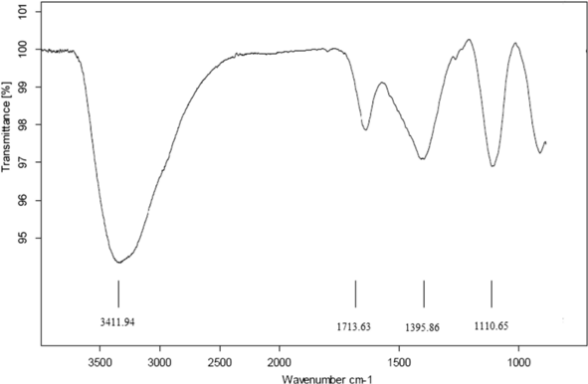
ATR-FTIR spectrum of synthesized GO

In addition to Raman spectra, IR spectrum is also used for GO characterization. Figure 3 shows the GO ATR-FTIR spectrum. From the figure, a prominent adsorption peak appeared at 3411.94 cm^-1^ that reveals the typical GO characteristics. This strong peak assigns to stretching vibration O-H bond and indicates the presence of hydroxyl groups. O-H bond may exist in forms of alcoholic, phenolic, carboxylic and so on. This peak also confirms the hydrophilic properties of GO. The band 1395.86 cm^-1^ can be attributed to O-H deformation vibration [21]. The absorption peak in the 1713.63 cm^-1^ shows the carbonyl stretching vibration (C = O) and indicate the presence of carboxyl functional group. In addition, appearance of an adsorption peak at 1110.65 cm^-1^ could be assigned to the C-O bond stretching vibration [38]. With regard to presence of oxygen containing functional groups it is proved that the synthesized GO is highly hydrophilic. These observations are consistent with the results reported in other works [12, 14].

### Characterization of the PSF/GO membrane

#### Effect of GO addition on membrane morphology

**Fig. 4:**
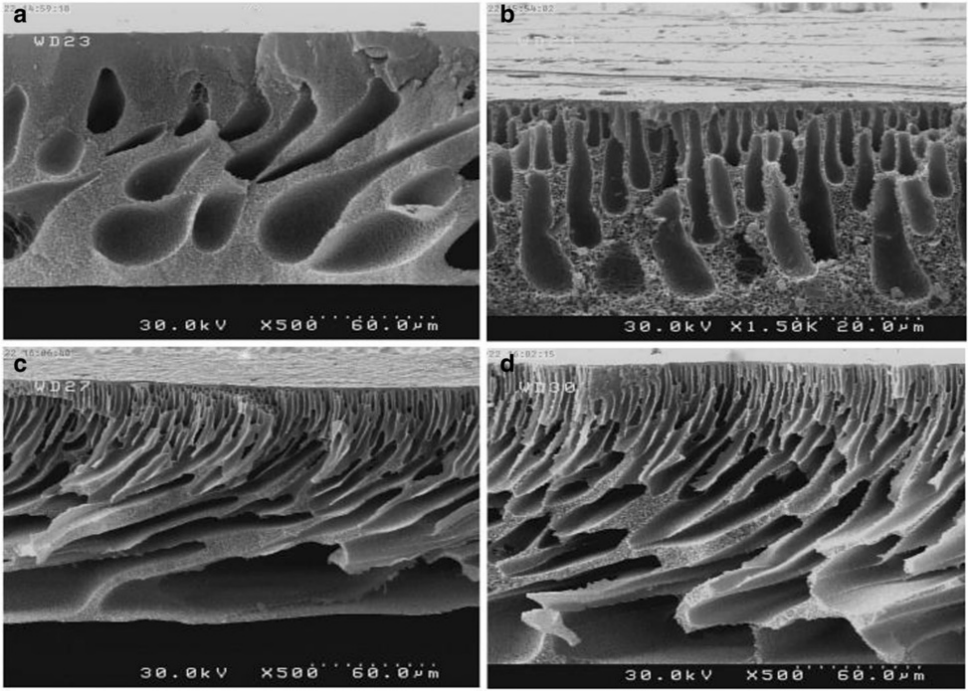
Cross-section morphologies FE-SEM images of the prepared membranes. **a** Pure PSF, **b** PSF/GO-0.5, **c** PSF/GO-1 and **d** PSF/GO-2 membranes

**Fig. 5:**
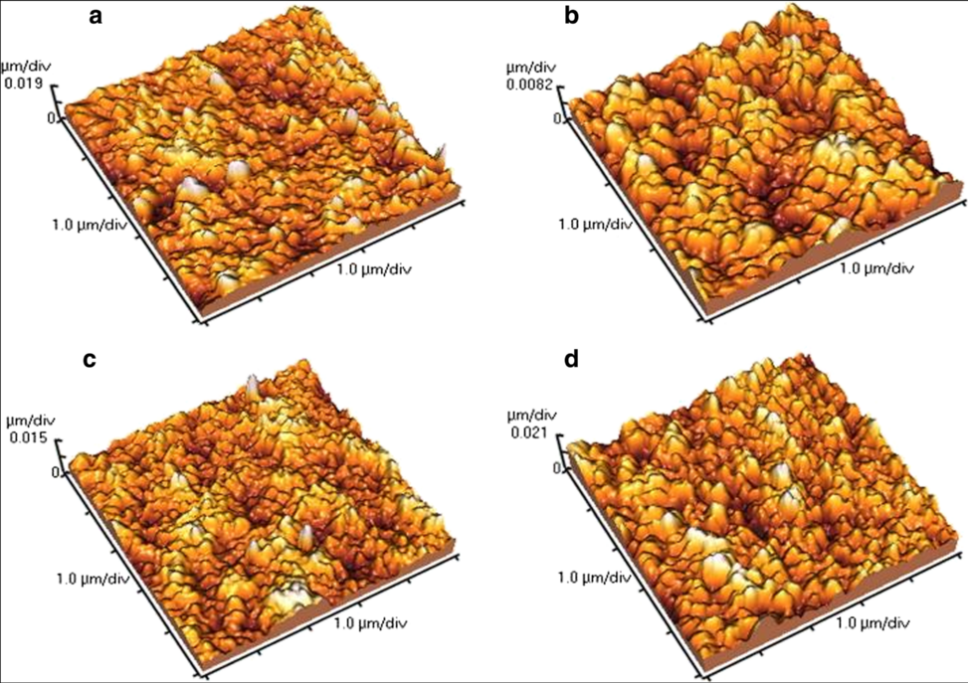
AFM three-dimensional surface morphology of the prepared membranes. **a** pure PSF, **b** PSF/GO-0.5, **c** PSF/GO-1 and **d** PSF/GO-2 membranes

In next stage of the study, the effect of loaded GO on the micro-structure of the PSF membrane was analyzed. Cross-sectional FE-SEM micrographs of the prepared membranes are presented in Fig. 4. General structure of the membrane consists of a dense skin layer on the top and a porous support sub layer. Pure PSF membrane with mainly sponge structure and few separated closed end drop-like pores shown in Fig. 4a. With the addition of GO, the main characteristics of a asymmetric structure appears composing of a dense skin layer on top and a thick porous layer with finger like pores in the bottom (Fig. 4b-d). From Fig. 4b, in membrane with 0.5 wt% GO, drop like pores have been replaced by finger-like pores in the pure PSF membrane but the walls of the pores are thick and with closed ends, and the sponge parts are still exist as a significant part of the membrane. With further increase in loaded GO, finger like channels turned into a large, open-end macrovoids and the spongy portion decreased significantly. Furthermore, from the figure, the number of their pores increased and walls thickness decreased compare to pristine PSF membrane (Fig. 4c-d). Generally, these structures have a low resistance to water permeation [39]. In addition, in the membranes with 1 and 2 wt% GO, horizontal channels appeared that can improve the water permeability. This issue is confirmed by other similar studies [14, 26]. The rate of pores production is the directly related to the exchange rate of solvent and non-solvent in the coagulation bath of phase inversion process. However, the faster the exchange rate of solvent and non-solvent in the coagulation process, the larger pores, more finger like pores and more channels. In contrast, the slower the exchange rate of solvent and non-solvent in the coagulation process, the smaller pores, more drop like pores and a spongy or non-void structure is resulted which finally alter the membrane permeability [40, 41]. By adding GO to the matrix of membrane the sub layer is effectively modified. This capability is attributed to GO hydrophilicity which results in thermodynamic instability in the casting solution, consequently rapid mass transfer between the solvent and nonsolvent is occurred. As a result, large pores are formed in the sub layer of membrane [5]. In this study, to evaluate the surface morphology of the synthesized membranes, AFM was used. In Fig. 5, three-dimensional images of the four types of synthesized membranes are illustrated. As it is obvious, the bright areas exhibit the highest points and dark areas depict the valleys or pores of the fabricated membranes. It seems that the direction of the dents is pointed to direction of applied coagulation bath. In addition, Table 1 presents the different roughness parameters of the membranes. From the Table, surface roughness of pure PSF membrane is greater than the modified membrane with 0.5 and 1 wt% GO, but it is less than membrane with 2 wt% GO. Adding a certain amount of GO changes the large peaks and valleys of the membrane to a large number of small peaks and valleys [26]. Actually, in low loading of carbon modifiers such as carbon nanotube and grapheme oxide, due to low electrostatic interaction and good compatibility with the membrane matrix, these nanomaterials could develop a suitable structure in the membrane, reducing the membrane roughness and thus create a smooth surface [42]. Similar behavior has been reported in previous studies [11, 43].

**Table 1:** Surface roughness parameters of the prepared membranes obtained from analyzing six randomly chosen surface AFM images

Membranes	Roughness parameters	
	Mean surface roughness (Ra-nm)	Root mean square roughness (Rq-nm)
Pure PSF	2.9 ± 0.23	3.9 ± 0.47
PSF/GO-0.5	2 ± 0.14	2.5 ± 0.15
PSF/GO-1	2.5 ± 0.30	3.4 ± 0.36
PSF/GO-2	4.4 ± 0.32	5.8 ± 0.50

### Membrane hydrophobicity, Water permeation flux and pore structure parameters

**Fig. 6:**
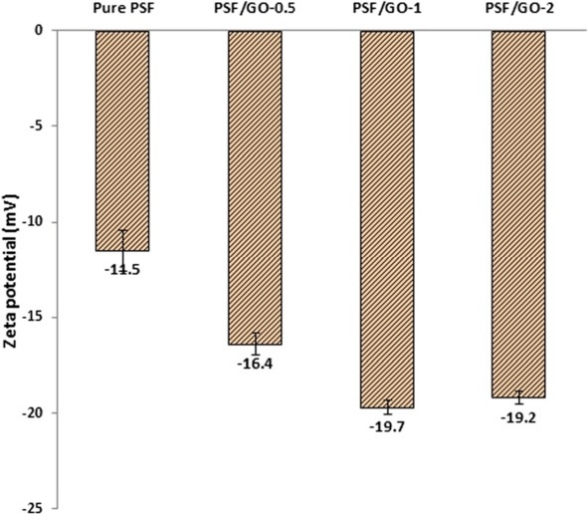
Surface zeta potential of the prepared membranes with various GO contents

Zeta potential values for synthesized membranes are presented in Fig. 6. As shown, all given values are negative. With increasing the amount of GO to 1 wt%, negatively charge and zeta potential increased. The results of contact angle measurements, porosity, pore size and water flux are given in Table 2. As shown in the table with an increase of 1 % GO nanoparticles to the polymer matrix, water contact angle decreased, in contrast the porosity, pore size and water flux increased. Accordingly, net PSF membrane has the highest contact angle, lowest values of porosity, pore size and flux. Among the membranes, PSF/GO-1 has the lowest contact angle and maximum porosity, pore size and water flux. From the table, PSF/GO-2 has slightly higher contact angle and lower pure water flux compare to PSF/GO-1 membrane. Generally, zeta potential plays an important role in flux and anti-fouling properties of membranes [44]. The surface charge is an indication of presence of charged functional groups on the membrane surface. Inducing of hydroxylic and carboxylic functional groups can produce negative charges on the membrane surface [20]. During the phase inversion process hydrophilic functional groups in GO migrate to the surface resulting in negatively charged surface. Blended nano particles in the membrane casting solution migrate to the top of the membrane that is initially exposed to the non-solvent (water) liquid. Increase of hydrophilic groups density on the membrane surface results in decrease of intermediate energy (interface energy) with water. As a result, with increasing the surface hydrophilicity, contact angle decreased [12]. The hydrophilic nature of GO speed up the exchange process of solvent and non solvent in the phase inversion method which increases the porosity and pore size of the membrane as clearly seen in FE-SEM micrographs (Fig. 4). These changes in the membrane properties enhance the membrane permeability [14, 45]. At GO contents of more than 1 wt%, (namely PSF/GO-2), the hydrophilicity of the membrane relatively reduced. This phenomenon may be due to accumulation and irregular positioning of GO nanoplates and decrease of the functional groups on the membrane surface. In addition, the reduction of water flux through membrane with GO content more than 1 wt%, is attributed to decrease of membrane porosity and pore size due to high viscosity of casting solution and delay of solvent and non-solvent exchange. In this situation, the pores are blocked by high concentrations of GO, resulting in flux reduction [11, 14]. The results of GO effects on the membrane characteristics are consistent with the similar works [12, 13, 21, 26].

**Table 2:** Effect of GO content on water contact angle, pure water flux and pore structure parameters of the prepared membranes

Membranes	Contact angle (deg)	Porosity (%)	Mean pore radius (nm)	Pure water flux (L/m^2^h)
Pure PSF	73.5 ± 2.1	48.3 ± 2.6	6.9 ± 0.56	19.7 ± 3.2
PSF/GO-0.5	66.7 ± 1.6	77.9 ± 2.2	8.3 ± 0.31	32.3 ± 3.5
PSF/GO-1	51.3 ± 1.2	86.5 ± 1.8	9.1 ± 0.63	49.9 ± 2.6
PSF/GO-2	54.8 ± 1.4	82.1 ± 1.3	8.7 ± 0.42	46.4 ± 2.0

### Arsenate rejection performance evaluation

**Fig. 7:**
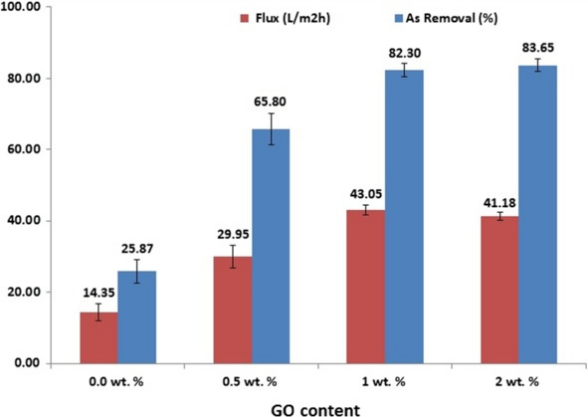
As(V) rejection and flux of the prepared membranes with various GO contents. (Operating pressure =4 bar, pH = 8.5 ± 0.2, Initial As(V) concentration = 300 ± 10 μg/L, feed temperature =25 ± 0.5 °C)

**Figure 8:**
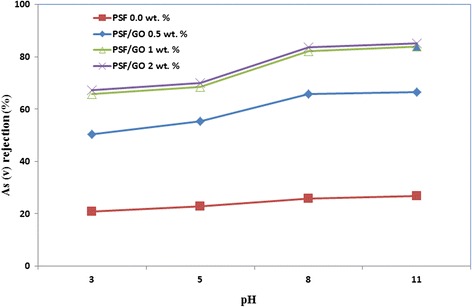
Percentage rejection of As (V) at different pH by prepared membranes with various GO contents. (Operating pressure =4 bar, Initial As (V) concentration = 300 ± 10 μg/L, feed temperature =25 ± 0.5 °C)

The results of arsenate rejection and membrane flux for different fabricated membranes are presented in Fig. 7. In the mentioned operating condition arsenate rejection for the pure PSF membrane and the modified membrane with 0.5, 1 and 2 wt% GO were 25.87 %, 65.80 %, 82.30 %, and 83.65 % respectively. The rejection by modified membranes are substantially higher than that of the pure PSF membrane. Moreover, by increasing the weight of GO in the casting solution the arsenate ions rejection increased. The reasons for this increase are described below. Negative hydrophilic functional groups such as hydroxyl and carboxyl groups on surface of GO can build up a high zeta potential by inducing negative charges on surface of the membrane. Negative charge of arsenate and negative charge on the membrane surface result in increase of Donnan repulsion, resulting in an increase in the arsenate rejection [46]. This feature does not exist in pure PSF membrane. Fundamentally, the charge repulsion of ions depends on the membrane charge, ionic strength and ions capacity [11]. Lohokare et al. reported that dominant removal mechanism of arsenate was Donnan exclusion using a modified hydrophilic UF membrane [46]. Moreover some researchers have previously proposed that modified hydrophilic membranes due to strong bonds with water can effectively prevent the passage of molecules [27]. However, it has been expressed that carbon nano-materials, could absorb foulants by surface reactions and consequently increase the rejection rate [32]. From Fig. 7, with increase in GO to 1 wt%, the increase in arsenate rejection is obvious, which is justified by the previously mentioned reasons. However, with increase to more than 1 wt% GO (PSF/GO-2) the removal efficiency is not very significant compared to the PSF/GO-1 membrane. This could be because of high density of irregular GO on membrane structure, reducing the functional groups on the membrane surface, resulting in decrease in membrane hydrophilicity [26]. Consequently with reduction of functional groups, negatively charged on membrane surface is reduced, thus the removal of arsenate does not increase proportion to loaded GO [11, 14]. In addition, a slight increase of arsenate rejection in PSF/GO-2 compare to PSF/GO-1 can be assigned to lower flux and pore size value. From Fig. 7, the results of flux of arsenate solution filtration for modified membranes showed an approximate 10 % reduction compared to pure water flux (Table 2), while the flux reduction is more in pure PSF membrane (about 27 %). This difference can be attributed to the nature of the hydrophilic and anti-fouling properties of the modified membranes. The rejection of arsenic by membrane could be affected by various parameters such as operating pressure, initial concentration, pH, ionic strength [46]. However, one of the most influential parameters is solution pH, which plays a major role in the rejection of arsenic by membrane systems [21, 47]. The effect of pH on the rate of arsenate rejection by synthesized membranes has been presented in Fig. 8. With increasing pH, the rejection increased due to some reasons. First, by increasing the pH, zeta potential of membrane increases and membrane surface charge becomes more negative [46, 48]. Moreover, arsenic charge which is controlled by pH, becomes more negative with increasing the pH [49]. Second, changing the pH values will change the predominant species of arsenic in the environment. So that at pH <6.9 monovalent ions (H_2_AsO_4_^-^) are dominant, while at pH > 6.9 divalent ions (HAsO_4_^2-^) are dominant. Therefore, with increasing pH, monovalent ions converted into divalent ions. Since the repulsive effect of Donnan is more dominant for divalent ions than monovalant ions, thus the rejection increased in higher pH values [47, 49, 50]. Accordingly Seidel et al. showed that the removal of arsenic by nanofiltration membranes was reduced from 85 % at pH = 8.5 to 8 % in pH = 4.5 [50]. Based on the good results of As (v) rejection obtained from the synthesized membrane, it seems that with determining the optimum operating parameters, the proposed standards for arsenic, especially in surface waters with the dominant species of arsenate, is achievable.

## Conclusion

In present study, GO nanoplate were directly added to PSF casting solution to fabricate a mixed matrix membrane via phase inversion method. The results showed that presence of abundant containing hydrophilic functional groups on GO, strongly enhance the hydrophilicity and permeability of the synthesized membrane. Graphene oxide also could modify the morphology of the membrane so that the spongy structure and closed-end drop like pores of the pure PSF membrane could change to finger like pores and larger open-end channels in PSF/GO membrane. Adding GO up to 1 wt% in casting solution resulted in enhancement of membrane morphology so that the contact angle reduced and the porosity and pure water flux increased due to the improvement of the membrane surface hydrophilicity. The results also showed that the rejection of arsenic in the PSF/GO membranes has substantially increased compared to pure PSF membrane. In addition, with increase in GO weight in the casting solution the rejection of arsenate ions increased. The experiments also showed that the predominant mechanism of arsenate rejection is, Donnan repulsion due to the negative charges induced by GO on the membrane surface. The results of this study revealed that due to unique properties of GO especially hydrophilicity, it can be considered as a promising nanomaterial for membrane fabrication and modification.
